# Single-cell metabolic profiling of stallion spermatozoa by flow cytometry using NADH and FAD autofluorescence^†^

**DOI:** 10.1093/biolre/ioaf294

**Published:** 2026-01-05

**Authors:** Laura Becerro-Rey, Francisco E Martín-Cano, Cristina Ortega-Ferrusola, Eva da Silva-Álvarez, Jose A Tapia, María Cruz Gil, Fernando J Peña

**Affiliations:** Laboratory of Equine Reproduction and Equine Spermatology, Veterinary Teaching Hospital, University of Extremadura, Cáceres, Spain; Laboratory of Equine Reproduction and Equine Spermatology, Veterinary Teaching Hospital, University of Extremadura, Cáceres, Spain; Laboratory of Equine Reproduction and Equine Spermatology, Veterinary Teaching Hospital, University of Extremadura, Cáceres, Spain; Laboratory of Equine Reproduction and Equine Spermatology, Veterinary Teaching Hospital, University of Extremadura, Cáceres, Spain; Department of Physiology, University of Extremadura, Cáceres, Spain; Laboratory of Equine Reproduction and Equine Spermatology, Veterinary Teaching Hospital, University of Extremadura, Cáceres, Spain; Laboratory of Equine Reproduction and Equine Spermatology, Veterinary Teaching Hospital, University of Extremadura, Cáceres, Spain

**Keywords:** stallion, spermatozoa, NAD(P)H, FAD, metabolism, flow cytometry

## Abstract

The metabolic activity of stallion spermatozoa was assessed using flow cytometry to detect NAD(P)H and FAD fluorescence without labels. Sperm were incubated with different energy sources—glucose, lactate, and pyruvate—individually or combined, and measurements of NAD(P)H, FAD, NAD(P)H/FAD ratio, and optical redox ratio (ORR) were taken. Additionally, a metabolic assay based on resazurin reduction and flow cytometry detection was developed. Changes in NAD(P)H and FAD fluorescence, the NAD(P)H/FAD ratio, and ORR were observed. The NAD(P)H/FAD ratio increased significantly, especially when glucose and pyruvate (*P* < 0.0001), glucose and lactate (*P* < 0.0001), or all three substrates (*P* < 0.0001) were present together. The mitochondrial activity index (MAI) and kinematic efficiency (KE) were also analyzed. Both indices showed positive correlations with the NADH/FAD ratio (0.6 and 0.7; *P* < 0.00001) and negative correlations with the ORR (−0.6 and − 0.7; *P* < 0.000001). Furthermore, flow cytometry was used to evaluate the spermatozoa’s ability to metabolize different substrates via resazurin reduction. Results indicated that stallion sperm preferentially oxidized glucose, lactate, and oxoglutarate. In summary, we developed a straightforward flow cytometry-based redox and metabolic assay. This, combined with the resazurin reduction test, revealed a preference for glucose and lactate oxidation. This suggests specialized metabolic compartmentalization, with glycolysis occurring in the flagella and oxidative phosphorylation in mitochondria. Additionally, this assay could be useful for clinical sperm assessment, as metabolic changes often reflect physiological alterations in sperm function.

## Teaser

Single-cell metabolic profiling of spermatozoa is achievable through flow cytometric quantification of NADH and FAD intrinsic autofluorescence.

## Graphical Abstract

**Graphical Abstract:**
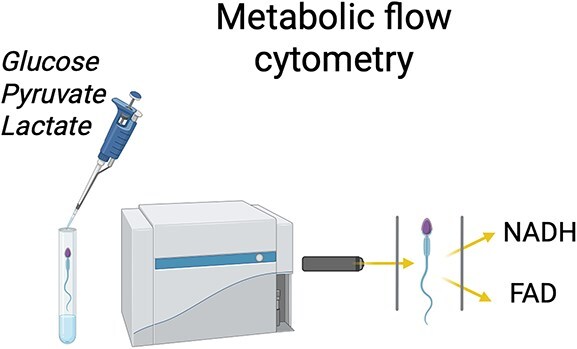

## Introduction

The complexity of mammalian ejaculate has long been recognized [1, 2]. Nevertheless, most studies and clinical assessments display methodological bias, frequently reducing it to a homogeneous entity rather than acknowledging its diverse cellular and molecular composition. Growing scientific evidence indicates that the ejaculate is composed of distinct groups of spermatozoa, probably with specific roles [3–5]. Only a few thousand complete the preparatory process of capacitation, and only one spermatozoon in the ejaculate finally fertilizes [4]. All this evidence underlines the need to assess the ejaculate following a single-cell approach. Another aspect that is receiving increased interest is sperm metabolism, which is being acknowledged as central to sperm biology and biotechnology [6–12]. The acquisition of fertilizing ability involves a biochemical adaptation of sperm metabolism to the increased energetic demands of capacitation [13, 14]. However, how the stallion spermatozoa respond to different metabolites is still poorly understood. Intense research in this area is warranted to improve current sperm biotechnologies and understand the characteristics of capacitation in horses [15]. Sophisticated methods to study sperm metabolism are required; however, these methods are expensive and located in specialized laboratories, such as those utilizing UHPLC/MS/MS or NMR technologies. Moreover, these methods do not provide information at the single sperm level.

Flow cytometry has been used for evaluating spermatozoa at the single-cell level for decades [16–21]. A promising method for measuring sperm metabolism at the single-cell level could be developed by leveraging the autofluorescence of nucleotides with important metabolic roles. These are the nicotinamide adenine dinucleotide (NADH) and flavin adenine dinucleotide (FAD). Glycolysis produces two moles of adenosine triphosphate (ATP) and two moles of NADH per mole of glucose metabolized. The enzyme glyceraldehyde phosphate dehydrogenase (GAPDH) plays a key role in this pathway, contributing to NADH generation. NADH is essential for reducing pyruvate to lactate and serves as a critical cofactor in lipid and glutathione synthesis, thereby supporting cellular redox homeostasis. In addition to being reduced to lactate, pyruvate may be transported into the mitochondria, where it is oxidized to acetyl-Coenzyme A (Acetyl-CoA). Acetyl-CoA enters the Krebs cycle, undergoing a series of energy-yielding reactions involving intermediates such as α-ketoglutarate, succinate, and fumarate. These reactions generate additional NADH and ultimately regenerate oxaloacetate, allowing the cycle to begin again with a new molecule of acetyl-CoA. The Krebs cycle, or tricarboxylic acid (TCA) cycle, is a fundamental metabolic pathway that underpins cellular respiration by generating reducing equivalents (NADH and FADH₂), which subsequently drive oxidative phosphorylation in mitochondria [22]. Within this cycle, succinate dehydrogenase (SDH)—designated as Complex II of the electron transport chain (ETC)—catalyzes the oxidation of succinate to fumarate, while simultaneously reducing FAD to FADH₂ [23]. This enzyme also facilitates the transfer of electrons from FADH₂ to ubiquinone (CoQ), reducing it to ubiquinol (CoQH₂), and contributes to the ETC without directly pumping protons across the inner mitochondrial membrane (IMM).Complex I (NADH: ubiquinone oxidoreductase) initiates the ETC by oxidizing NADH and transferring electrons to ubiquinone. It also translocates protons from the mitochondrial matrix to the intermembrane space, thereby establishing the proton motive force. During this process, NADH is oxidized to NAD^+^, a step also mirrored in lactate generation from pyruvate. Complexes I, III, and IV of the ETC actively translocate protons across the IMM, establishing the electrochemical gradient essential for ATP synthesis [24]. Complex IV (cytochrome c oxidase) completes the electron transfer chain by reducing molecular oxygen to water. Finally, Complex V (ATP synthase) utilizes the chemiosmotic gradient generated by proton pumping to drive the phosphorylation of ADP to ATP. Consequently, the redox state of cells, defined as the ratio of reduced to oxidized NADH and FAD, can serve as a proxy for metabolic status. The reduced form, NADH, is fluorescent upon excitation at 340–355 nm [1, 16], with an emission maximum around 440–470 nm. FAD has an excitation maximum at 450–470 nm and an emission maximum at 520–535 nm. Using 360 nm excitation for NADH and 405 nm for FAD provides improved redox resolution. NADH exhibits a strong absorption maximum at ~340 nm (ε ≈ 6200 M^−1^ cm^−1^) with ~90–100% relative efficiency in the 339–365 nm range, whereas absorption falls off dramatically by 405 nm (<5% of peak). Conversely, FAD features two absorption bands around 368 nm and 450 nm, with 405 nm excitation lying on the tail of both, yielding approximately 20–30% of its peak absorption at 450 nm [25]. Thus, the 360/405 nm combination achieves highly selective excitation: NAD(P)H is efficiently stimulated at ~360 nm with minimal FAD crosstalk, and FAD is selectively excited at 405 nm with negligible NAD(P)H overlap. This ensures superior fluorophore separation and minimal spectral bleed-through—ideal for redox imaging. The fluorescence of NAD^+^ and FADH_2_ is much lower and typically undetectable. Thus, the ratio NAD(P)H/FAD or redox ratio (RR) may reflect the metabolic and redox state [26–28], as does the optical redox ratio (ORR) of FAD/[NAD(P)H + FAD] across multiple cell types and tissues [29]. The RR and ORR, therefore, provide non-invasive indicators of cellular metabolic states at the single-cell level. Recently, label-free measurement of NAD(P)H and FAD by flow cytometry has been used to distinguish immune cell populations and glioma cells [30]. Immune cells activated by specific stimuli show increased NAD(P)H, and different purified immune cell types exhibit distinct redox ratios. We investigated sperm responses to acute metabolic challenges, including incubation of glucose, lactate, and pyruvate, singly or in combination, OXPHOS inhibition, and mitochondrial uncoupling. We applied flow cytometry to quantify changes in NADH, FAD, the redox ratio (RR), and the ORR. This label-free approach revealed fluorescence shifts in response to metabolic perturbations, enabling single-cell analysis of sperm metabolism and supporting its potential for sperm selection and functional evaluation. Our findings further show that stallion spermatozoa preferentially oxidize glucose and lactate, with redox balance maintained through LDH-mediated reduction of pyruvate to lactate, sustaining the NADH/NAD^+^ pair.

## Material and methods

### Reagents and media

Chemicals were purchased from Sigma Aldrich (Madrid, Spain) Tetramethylrhodamine, Methyl Ester. (TMRM) was purchased from Thermo Fisher (Carlsbad, Ca USA). ViaKrome 808 Fixable Viability Dye was purchased from Beckman Coulter (Indianapolis, IN USA). Ultra-pure deionized water (>18.2 MΩ·cm) was produced from a Millipore Milli-Q Gradient system (Millipore, Bedford, MA, USA).

### Semen collection and processing

Semen was collected from four fertile stallions maintained according to institutional and European animal care regulations (Law 6/2913, June 11th and European Directive 2010/63/EU). Ejaculates were collected using a pre-warmed, lubricated Missouri model artificial vagina following standard veterinary practices. After collection, the ejaculate was immediately evaluated for sperm motility and concentration and processed in the adjacent laboratory. Spermatozoa were extended to 25 x 10^6^ spermatozoa/ml in phosphate-buffered saline (PBS) or Tyrode’s media containing different sources of energy (glucose, pyruvate, lactate), alone or in combination, depending on the specific experiment. The mitochondrial uncoupler FCCP (1, 2, and 5 μM), the ATP synthase inhibitor oligomycin (10, 20, and 30 μM), and the complex I inhibitor Rotenone (1 μM) were used in specific experiments. The pH was adjusted to 7.4, and the osmolarity to 310 mOsm/kg.

### Experimental design

A split-sample design was employed, whereby each ejaculate was subdivided and allocated to the different experimental conditions, ensuring that all treatments were applied to aliquots derived from the same ejaculate and stallion. Individual ejaculates were split and extended to a concentration of 25 × 10^6^ spermatozoa per mL in different media. PBS, Tyrode’s media [31], and the different variations, including media devoid of any energy source, media including glucose, pyruvate, or lactate as an energy source, and other combinations of energy sources depending on the specific experiment. Samples were incubated at 37°C for 2 h, and then aliquots were taken for sperm analysis. The parameters studied were the following: motility and kinematics (CASA) and metabolic status using flow cytometry. Additional experiments uncoupling the mitochondria with Carbonyl cyanide 4-(trifluoromethoxy)phenylhydrazone (FCCP) to induce a compensatory increase in mitochondrial respiration, and using rotenone to inhibit complex I in the ETC, not allowing the release of electrons from NADH, were performed.

### Computer-assisted sperm analysis

Sperm motility and kinematics were assessed using a Computer Assisted Sperm Analysis (CASA) system (ISAS Proiser, Valencia, Spain) according to standard protocols used at our center [32]. Semen samples were loaded in a Leja chamber with a depth of 20 μm (Leja, Amsterdam, The Netherlands) and placed on a stage warmed to 37°C. The analysis was based on an evaluation of 60 consecutive digitized images, with a frame rate of 60 Hz, obtained using a 10x negative phase-contrast objective (Olympus CX 41). At least 500 spermatozoa per sample were analyzed in random fields. Spermatozoa with average velocity (VAP) >35 μm/s were considered motile. Spermatozoa deviating <45% from a straight line were classified as linearly motile. Other parameters studied included curvilinear velocity (VCL μm/s), defined as the time-averaged velocity of a sperm head along its actual trajectory, the straight-line velocity (VSL μm/s), the velocity calculated along a straight line between the first and last points of the path, and velocity along the average path (VAP μm/s), as the time-averaged velocity calculated along the average path. Additionally, kinematic efficiency (KE), the coefficient between the curvilinear velocity and the beat cross frequency (VCL/BSF), was calculated as described in [33]; this parameter is the average point-to-point distance along the sperm path in micrometers, traveled per cross of the sperm head across the path. This parameter is considered a better indicator of the metabolic status of the spermatozoa.

### Flow cytometry

Flow cytometry was conducted according to published guidelines [34] adapted to spermatozoa. The analyses were performed using a Cytoflex LX equipped with ultraviolet (355 nm), violet (405 nm), blue (488 nm), yellow-green (561 nm), red (638 nm), and infrared (808 nm) lasers. The instrument was calibrated daily using specific calibration beads provided by the manufacturer. Data were exported as FCS files, and then compensated and analyzed in Cytobank Software (Beckman Coulter, Brea, CA, USA). Unstained, single-stained, and Fluorescence Minus One (FMO) controls, when applicable, were used to determine compensations and positive and negative events, as well as to set regions of interest as described in previous publications by our laboratory [35, 36], following the recommendations published as minimum information about a flow cytometry experiment standard (MyFlowCyt) [37].

### Label-free determination of NADH and FAD fluorescence in individual stallion spermatozoa

**Figure 1:**
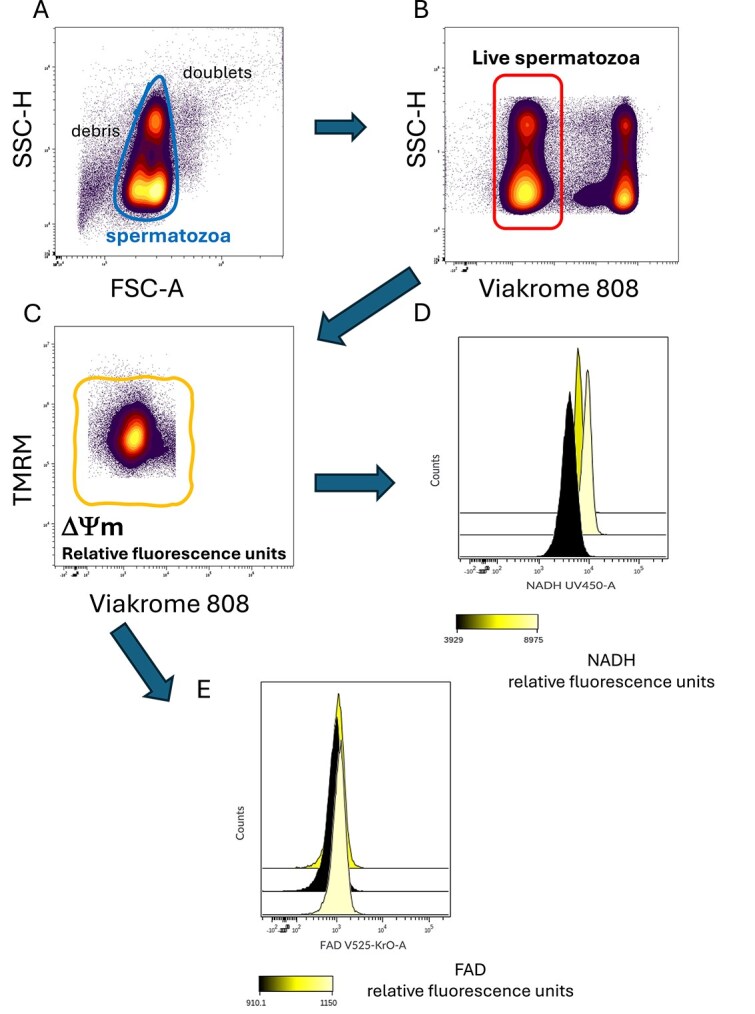
Gating strategy of the flow cytometry metabolic assay. Stallion spermatozoa were processed and stained for flow cytometry analysis as described in Materials and methods. (A) Spermatozoa were identified based on FSC and SCC characteristics. Doublets, clumps, and debris were gated out. (B) Dead spermatozoa were removed from the analysis using a viability dye (Viakrome 808), live, membrane-intact spermatozoa were gated, and further analysis was conducted in this population. (C) Mitochondrial membrane potential (D) NAD(P)H fluorescence (E) FAD fluorescence.

NADH fluorescence was measured following excitation at 355 nm and detection at the emission maximum of 488 nm using a 450/20 BP filter. FAD fluorescence was assessed after excitation at 405 nm and detection with a 550/40 BP filter, following previously published protocols [30, 33, 38]. Simultaneously, cell viability was evaluated using ViaKrome 808 (Beckman Coulter, C36628; 2 μL/sample; Ex/Em 854/878 nm) and mitochondrial electrical and diffusive potential (membrane potential) was determined with tetramethylrhodamine methyl ester (TMRM; ThermoFisher, I34361; 50 nM; Ex/Em 548/574 nm) In spermatozoa, TMRM fluorescence localized specifically to the mitochondria (Supplementary Figure S1) The mitochondrial activity index (MAI) was calculated based on [39], and defined as the median fluorescence intensity (relative fluorescence units, r.f.u.) of live spermatozoa exhibiting high mitochondrial potential, normalized to the total number of spermatozoa within the gate. This parameter serves as a proxy for the mitochondrial membrane potential of individual spermatozoa. The gating strategy is illustrated in Figure 1. To validate the TMRM assay, controls included the protonophore FCCP (uncoupling agent) and oligomycin (ATP synthase inhibitor). Fluorescence localization was confirmed by image flow cytometry as previously described [31], using an ImageStream X Mark II Imaging Flow Cytometer (Merck Millipore) using a laser of 542 nm line with intensity set to 100 mW, at 60X of magnification. Data analysis of the raw images was accomplished using IDEAS1software (Version 6.0.309). Since TMRM may exhibit different behavior depending on the concentration used [40, 41], titration experiments were performed to determine the optimal TMRM concentration for stallion spermatozoa. The assay was validated using the uncoupler FCCP (1, 2, and 5 μM), and ATP synthase inhibitor oligomycin (10, 20, and 30 μM) (Supplementary Figure S1).

### Flow cytometry–based metabolite oxidation assay

This test is based on previously published protocols [33, 42] adapted to flow cytometry for the stallion spermatozoa. This test is based on the measurement of mitochondrial NADH reductase and other diaphorase activity, including dehydrogenases and cytochrome reductases, through the reduction of the cell-permeable and non-fluorescent compound resazurin to the fluorescent compound resorufin. The intensity of resorufin fluorescence is proportional to the ability of stallion spermatozoa to incorporate and oxidize extracellular catabolites. Each of the catabolites studied was added independently. Stallion spermatozoa 25x10^6^ in 500 μL were extended in Tyrode’s media without energy sources (negative control), and variants of this media containing 5 mM glucose, 5 mM fructose, 5 mM lactose, 5 mM lactate, 5 mM pyruvate, and 5 mM oxoglutarate. After incubation at 37°C for 30 and 60 min in the presence of resazurin 80 μM, aliquots were taken for flow cytometry analysis. The reduction of resazurin to resorufin was monitored in a Cytoflex LX flow cytometer, using the yellow laser excitation (561 nm) and emission at 590 nm PE band pass filter (585/15).

### High-dimensional analysis of flow cytometry data

FlowSOM (Flow Self-Organizing Maps) is a computational tool used in flow cytometry to analyze high-dimensional data. It combines clustering and visualization to group sperm cells into distinct populations based on their marker expression (in this case, NAD(P)H and FAD fluorescence). FlowSOM uses a self-organizing map (SOM) to reduce the data into a grid of nodes, where each node represents a cluster of cells with similar characteristics. These clusters are then further refined using a hierarchical consensus method, allowing researchers to identify and visualize subpopulations of sperm, such as those undergoing capacitation or showing signs of apoptosis, or, as in this case, in different metabolic states. FlowSOM is efficient, scalable, and particularly useful for uncovering rare cell populations or subtle differences in sperm function, making it a valuable tool for detailed sperm analysis in flow cytometry [18, 19, 43–45]. Flow cytometry data were analyzed using the FlowSOM algorithm implemented in Cytobank (Beckman Coulter Life Sciences) with default settings. File-internal compensation was applied, and data were normalized before clustering. Four channels (FL1-A, FL13-A, FL18-A, FL21-A) were used for clustering. A total of 100,002 events were sampled proportionally across 36 FCS files. FlowSOM clustering was performed using the standard two-step workflow consisting of SOM training followed by hierarchical metaclustering. The SOM was initialized with 100 clusters, a commonly used default that provides sufficient granularity to capture the continuous variation in NADH and FAD autofluorescence intensities that define metabolic heterogeneity in spermatozoa. Each cluster represents a prototype phenotype within the multidimensional fluorescence space, enabling fine-resolution mapping of redox-related metabolic states without over-fragmenting the data. Following SOM training, the clusters were aggregated into 10 metaclusters using consensus hierarchical clustering. This number of metaclusters offers an effective balance between resolution and interpretability, allowing broader metabolic states to emerge from the finer SOM structure. Exploratory analyses using alternative SOM sizes and metacluster numbers yielded comparable topologies and did not alter downstream biological interpretations, supporting the robustness of these parameter choices.

FlowSOM does not apply explicit weighting to clusters; instead, cluster sizes reflect the number of events assigned to each node in the SOM. The random seed was automatically generated by Cytobank (42144023). Cluster visualization was performed with relative sizing in plots, and populations were labeled according to experimental conditions.

### Statistical analysis

All experiments were repeated at least three times with independent biological replicates. The normality of the data was assessed using the Kolmogorov–Smirnov test. One-way ANOVA followed by Tukey multiple comparisons test was performed using GraphPad Prism version 10.3.0 for Mac, La Jolla, California USA, www.graphpad.com

## Results

### Effect of different single energy sources on NADH, FAD, NADH/FAD ratio, and the ORR in stallion spermatozoa

**Figure 2:**
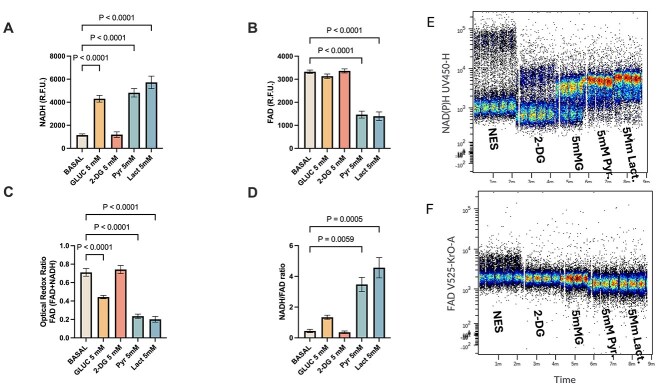
Effect of individual metabolic substrates on NADH and FAD fluorescence in stallion spermatozoa. Ejaculates from four different stallions were collected and processed as described in materials and methods and extended to 20x10^6^ spermatozoa/ml in PBS in the absence of energy sources (Basal) or in the presence of 5 mM glucose, 5 mM 2-deoxyglucose, 5 mM pyruvate or 5 mM lactate. NADH and FAD were determined using flow cytometry. (A) Changes in NADH fluorescence (B) Changes in FAD fluorescence (C) Changes in the optical redox rate (ORR), (D) Changes in the NADH/FAD rate (E) Time versus NADH fluorescence plot showing changes in florescence in presence of different metabolites (F) Time versus FAD fluorescence plot showing the effect of different metabolites. (A) a-b-c *P* < 0.0001; b-c *P* < 0.01. Results are derived from 12 identical experiments (four horses three ejaculates each).

Stallion spermatozoa were extended in a basal medium (phosphate-buffered saline) and supplemented with different energy sources: glucose 5 mM, pyruvate 5 mM, and lactate 5 mM. After 2 h of incubation at 38°C, spermatozoa were evaluated using flow cytometry. NAD(P)H and FAD fluorescence were evaluated, and the NAD(P)H/FAD and ORR were calculated. The addition of energy sources increased NAD(P)H fluorescence (Figure 2A, *P* < 0.0001), with fluorescence in the presence of glucose 5 mM, pyruvate 5 mM, and lactate 5 mM being significantly higher than in the absence of energy sources or the presence of the non-metabolizable glucose analog 2-deoxyglucose (2DG). Fluorescence of the reduced form of FAD was diminished in the presence of 5 mM pyruvate and 5 mM lactate, compared with the aliquots incubated in the absence of energy sources, 5 mM glucose, and 5 mM 2-DG (Figure 2B; *P* < 0.0001). The ORR was also affected by the energy source in the media, with higher ORR in the basal (no source of energy) and 2-DG glucose media (Figure 2C; *P* < 0.0001). The NADH/FAD ratio was also affected by the energy source present, being higher in the presence of pyruvate and lactate (Figure 2D, *P* < 0.01 and *P* < 0.001, respectively).

### Effect of different energy sources, and their combinations, on stallion sperm motility, kinematics, mitochondrial electrochemical gradient, and viability.

**Figure 3:**
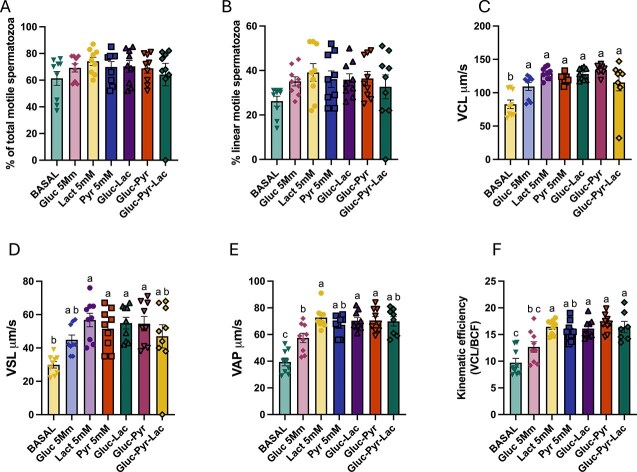
Effect of different metabolites and their combinations on stallion spermatozoa incubated for 2 h are 37°C in Tyrode’s media. Stallion ejaculates were processed as described in materials and methods and extended to 20 x10^6^ spermatozoa in Tyrode’s media, with or without different metabolites and their combinations at 37°C in aerobiosis for 2 h. Basal = no exogenous energy source, Glucose 5 mM, lactate 5 mM, pyruvate 5 mM, Glucose 5 mM-lactate 5 mM, Glucose 5 mM-Pyruvate 5 mM, and Glucose 5 mM- pyruvate 5 mM Lactate 5 mM. After the incubation period, samples were taken for analysis of sperm kinematics using a computer-assisted sperm analysis system (CASA). Parameters studied included (A) the percentage of total motile spermatozoa, (B) the percentage of linear motile spermatozoa, (C) VCL circular velocity in μm/s, (D) VSL straight line velocity in μm/s, (E) VAP, average velocity in μm/s, (F) KE kinematic efficiency, the average point-to-point distance along the sperm path in micrometers, traveled per cross of the sperm head across the path. Results are derived from four different stallions four replicates each (n = 12). VCL a-b *P* < 0.01, VSL, a-b *P* < 0.0001, VAP a-b *P* < 0.01, a-c *P* < 0.0001, c-b *P* < 0.001, KE, a-c *P* < 0.0001 a-b *P* < 0.01

**Figure 4:**
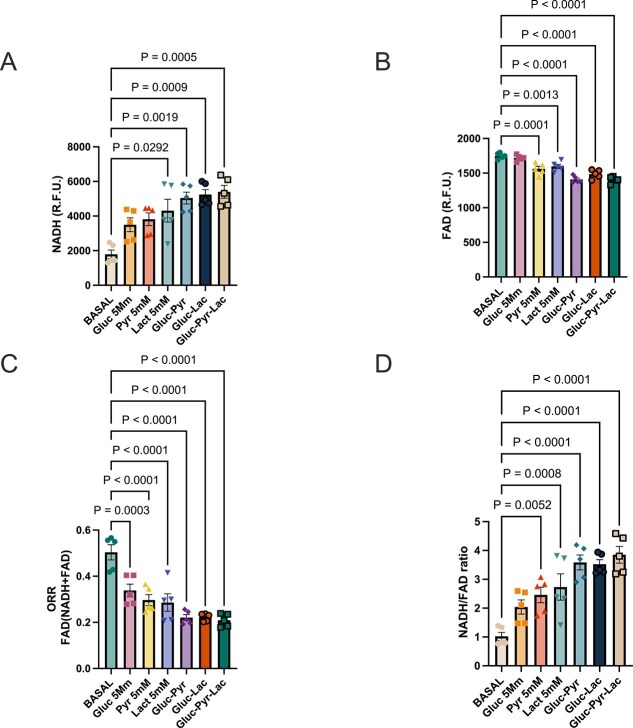
Effect of different metabolites and their combinations on stallion spermatozoa incubated for 2 h at 37°C in Tyrode’s media. Stallion ejaculates were processed as described in Materials and methods and extended to 20 x10^6^ spermatozoa in Tyrode’s media, with or without different metabolites and their combinations at 37°C in aerobiosis for 2 h. Basal = no exogenous energy source, Glucose 5 mM, lactate 5 mM, pyruvate 5 mM, Glucose 5 mM-lactate 5 mM, Glucose 5 mM-pyruvate 5 mM, and Glucose 5 mM-pyruvate 5 mM-lactate. After the incubation period, samples were taken for label-free flow cytometric determination of changes in NADH and FAD fluorescence. (A) Changes in NADH fluorescence, note that glucose and pyruvate did not increase NADH over basal values, changes were observed in the presence of lactate as the single exogenous energy source, and in combinations of glucose, lactate, and pyruvate (B) Changes in FAD fluorescence (C) Optical Redox Rate (ORR) (D) NADH/FAD rate. Results are derived from five different horses, three ejaculates each.

A new experiment was conducted to determine if the changes observed in NAD(P)H and FAD fluorescence, along with the NAD(P)H/FAD ratio and ORR, are linked to variations in sperm functionality. Additionally, the effects of various energy sources in a balanced salt-based medium were examined. The percentages of total and linear motile sperm remained stable regardless of the energy source present. Also, when no energy source was added to the media during the 2-h incubation, motility stayed consistent (Figure 3A and B). Sperm velocities were affected by the energy source in the media (Figure 3C–E, *P* < 0.01), as was KE; the lowest KE was observed in media without energy sources (Figure 3F; *P* < 0.0001), while higher KE levels were found in media containing lactate and pyruvate, with or without glucose, compared to media with only glucose as the sole exogenous energy source (Figure 3F, *P* < 0.01). The percentages of viable, membrane-intact spermatozoa were unaffected by the presence or absence of energy sources or the type of energy source (Figure 4A). The energy source also influenced mitochondrial membrane potential. It is worth noting to remember that strictly speaking, potentiometric dyes such as TMRM report changes in the electrochemical gradient across the IMM, which reflects both electrical and chemical (proton) components. Neither glucose nor pyruvate alone increased mitochondrial activity, but lactate (*P* = 0.0023), the combination of glucose and pyruvate (*P* = 0.0052), glucose and lactate (*P* = 0.0080), and glucose, pyruvate, and lactate (*P* = 0.0202) all elevated the MAI (Figure 4B).

### The response to lactate and pyruvate is different among sperm subpopulations

**Figure 5:**
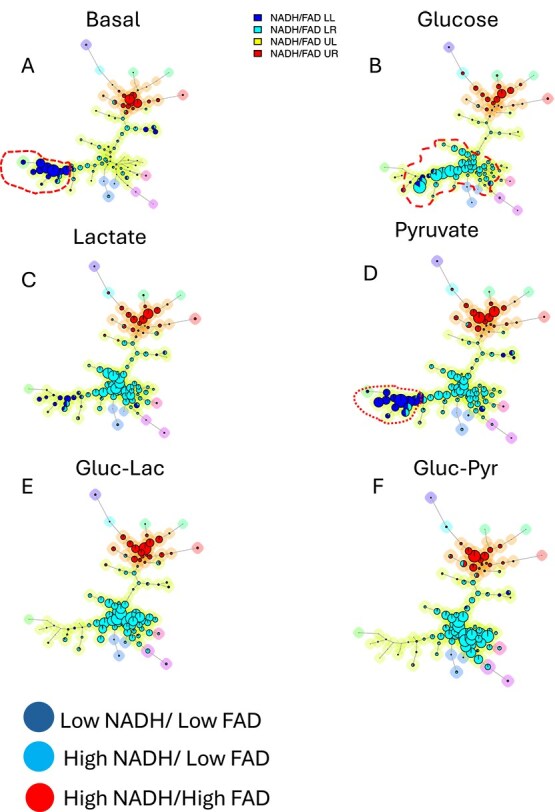
FloSOM cluster analysis. FlowSOM is an algorithm based on a self-organizing map (SOM), a type of unsupervised self-learning algorithm that can identify patterns and group data without requiring predefined categories or labels. Stallion spermatozoa were processed as described in the Materials and Methods section, and NADH and FAD fluorescence in the presence of different energy sources was measured using a label-free flow cytometry protocol. (A) Self-organizing map (SOM) in the absence of exogenous sources of energy, the predominant population of spermatozoa was cells showing low NADH and FAD fluorescence (navy blue, within the red dot region). (B) In the presence of glucose, the mentioned population virtually disappeared, and the predominant population was the spermatozoa with high NADH and low FAD fluorescence. C and D show the self-organizing maps in the presence of lactate and pyruvate; the most evident difference is the more homogeneous response in the presence of lactate, in which the population of low NADH and FAD fluorescence was predominant, while in the presence of pyruvate and low NADH and FAD fluorescence remained, (D red dots population). E and F, show the SOM map when glucose and lactate (E) or pyruvate (F) are present simultaneously, under these conditions the predominant population is the spermatozoa showing high NADH and FAD fluorescence.

To study whether the response to the presence of different energy sources was homogeneous among all the spermatozoa present in the ejaculate, the redox shift was studied using conventional and computational flow cytometry. Addition of various energy sources induced a shift to a more reduced state however, this shift was higher for lactate, in which the response was evident in all the spermatozoa (Supplementary Figure S1C), on the contrary, pyruvate produced a dual response, with a responsive population and a population of spermatozoa showing no displacement to a more reduced state (Supplementary Figure S2D). The combinations of glucose and pyruvate or lactate induced a shift to a more reduced redox state in the whole population of spermatozoa (Supplementary Figure S2E and F). The FlowSOM algorithm was used for detecting specific subsets of spermatozoa [45]. In the absence of energy sources, the predominant population of spermatozoa was cells with low NADH and FAD, When different individual sources of energy were added, the population structure changed and the main population of spermatozoa showed a shift to a more reduced redox state (Figure 5), however when pyruvate was the only source of energy, a population in a more oxidized redox state was evident (Figure 5D). The combination of glucose and pyruvate, and of glucose and lactate, caused a major shift toward a more reduced redox state (Figure 5E and F).

### Effect of different sources of energy and their combinations on NADH, FAD, NADH/FAD ratio, and the ORR in stallion spermatozoa

**Figure 6:**
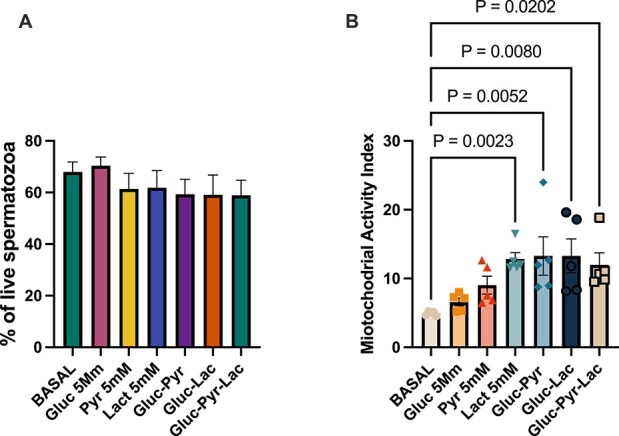
Effect of the energy source present in the media on stallion sperm membrane integrity and mitochondrial function. Stallion spermatozoa were processed as described in the Materials and Methods section, and viability and mitochondrial activity in the presence of different sources of energy were measured using flow cytometry. Viability was measured using the fixable viability probe Viakrome 808 as a percentage of viable spermatozoa. Mitochondrial function was calculated using the TMRM dye, creating a gate in the population of live spermatozoa showing high membrane potential. Then fluorescence intensity was measured in this region (RFU), and normalized to the number of spermatozoa found in the gate. This normalization provides a proxy of the mitochondrial activity of every individual spermatozoa and was named as Mitochondrial activity index (MAI). (A) The source of energy did not affect sperm viability. (B) The mitochondrial activity index was influenced by the energy source (s) present in the media, with lactate and the combination of glucose with lactate and or pyruvate significantly increasing mitochondrial activity. Results are derived from five different horses, three ejaculates each.

Stallion spermatozoa were extended in a Tyrode’s basal medium and supplemented with different energy sources, singly or in combination: no exogenous energy source, glucose 5 mM, pyruvate 5 mM, lactate 5 mM, glucose + lactate, glucose + pyruvate, and glucose + lactate + pyruvate. After 2 h of incubation at 38°C, spermatozoa were evaluated using flow cytometry. Dead spermatozoa were gated out after staining with a viability dye (Viakrome 808), then NADH and FAD fluorescence were assessed, and the NADH/FAD ratio and optical redox rate (ORR) were calculated in the viable population of cells. The lowest NADH fluorescence corresponded to the aliquot incubated without an energy source and increased in the aliquots in which an energy source was present. Significant differences appeared in the presence of lactate (*P* = 0.0292) and combinations of glucose and pyruvate (*P* = 0.0019), glucose and lactate (*P* = 0.0009), and glucose, lactate, and pyruvate (*P* = 0.0005; Figure 6). The FAD fluorescence followed a similar pattern, and more significant differences were observed in the aliquots in which a combination of energy sources was present (Figure 6B; *P* < 0.0001). The ORR showed more evident changes in the presence of energy sources in the media, with evident changes in the presence of energy sources, although the more evident changes occurred in presence of combinations of energy sources (Figure 6C; *P* < 0.0001). Finally, the NADH/FAD ratio does not increased in the presence of glucose, but did it in presence of all other sources of energy, and especially in the presence of glucose and pyruvate (*P* < 0.0001; Figure 6D), glucose and lactate (*P* < 0.0001; Figure 5D), and glucose, pyruvate an lactate (*P* < 0.0001; Figure 6D).

### The NADH/FAD ratio and ORR correlate with sperm functionality

**Figure 7:**
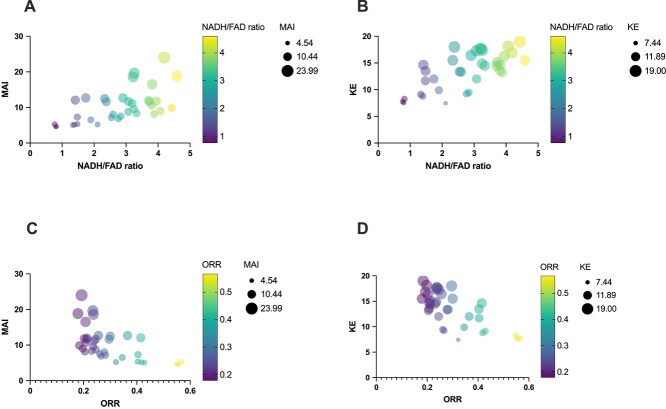
Bubble plots showing the correlations among kinematic efficiency (KE) and mitochondrial activity index (MAI), the NADH/FAD ratio, and the optical redox ratio (ORR). MAI and KE were positively correlated with the NADH/FAD ratio (0.6 and 0 7; *P* < 0.00001), respectively, while both parameters were negatively correlated with the ORR (−0.6 and − 0.7; *P* < 0.000001). Results are derived from five different horses, three ejaculates each.

Correlations among KE and MAI, the NADH/FAD ratio, and the ORR were investigated. Both indexes were positively correlated with the NADH/FAD ratio (Figure 7A and B; 0.6 and 0 7; *P* < 0.00001), respectively, while both parameters were negatively correlated with the ORR (Figure 7C and D; −0.6 and − 0.7; *P* < 0.000001).

### Effect of uncoupling mitochondria and disrupting the electron transport chain on NADH, FAD, and ORR

If most of NADH is produced during glycolysis and the TCA cycle and consumed in the ETC, then uncoupling mitochondria shall decrease NADH fluorescence, because the ETC speeds up to compensate for the lost proton gradient, rapidly oxidizing NADH to NAD^+^. Conversely, blocking the ETC increases NADH because the electrons can no longer be passed along, causing NADH to accumulate as it cannot be converted back to NAD^+^. Inhibition of NADH-linked mitochondrial respiration with rotenone and collapsing the mitochondrial membrane potential (ΔΨm) with the mitochondrial uncoupler FCCP was used to provide further confirmation that changes in NADH and FAD can be detected using flow cytometry in stallion spermatozoa. Semen samples were incubated in the presence of FCCP (1–5 μM) and the inhibitor of complex I of the ETC, rotenone (1 μM). As hypothesized, inhibition of the complex I induced a significant increase in NADH fluorescence (Supplementary Figure S3A; *P* = 0.0241). To control possible intrinsic rotenone autofluorescence a control consisting of rotenone in the absence of energy sources was included. While uncoupling the mitochondria caused a substantial reduction in NADH fluorescence (Supplementary Figure S3B; *P* = 0.0004). The NADH/FAD and ORR also showed the expected changes with a shift to a more reduced state after inhibition of the electron transport (Supplementary Figure S3D; *P* < 0.0001) and to a more oxidized state after mitochondrial uncoupling (Supplementary Figure S3C; *P* < 0.0001).

### Stallion spermatozoa preferentially oxidize glucose and lactate

**Figure 8:**
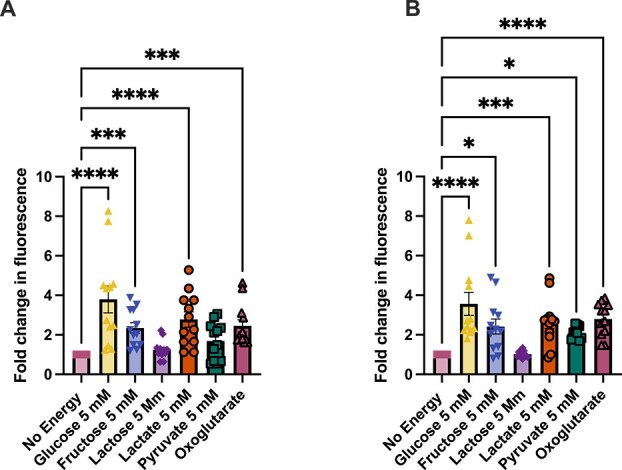
Stallion spermatozoa preferentially oxidize glucose and lactate. To estimate the ability of stallion spermatozoa to take up and oxidize different metabolites, a flow cytometry-based assay was developed. This assay measured sperm diaphorase activity (e.g. mitochondrial NADH oxidoreductase) as the cell-permeant and non-fluorescent resazurin is reduced to the highly fluorescent resazurin [33]. Controls non including resazurin, and controls including the dye but not spermatozoa, were included. The fold change in fluorescence in relative fluorescence units (RFU) with respect the sample with no energy sources in the media is reported as means ± SE, derived from four horses three replicates each. ^*^^*^^*^^*^P<0.0001, ^*^^*^^*^P<0.001, ^*^^*^P < 0.01,^*^P < 0.05.

The ability of stallion spermatozoa to incorporate and oxidize different metabolites was measured using a flow cytometry-based assay, adapted from previously published protocols as described in materials and methods. After 30′ of incubation, stallion spermatozoa oxidized glucose (*P* < 0.0001), fructose (*P* < 0.001), lactate (*P* < 0.0001), and alpha-ketoglutarate (*P* < 0.001), but not pyruvate. After 60 min, stallion spermatozoa oxidized glucose (*P* < 0.0001), lactate (*P* < 0.001), and alpha-ketoglutarate (*P* < 0.0001); fructose and pyruvate were also oxidized after 60′ of incubation (*P* < 0.05). Lactose were not oxidized by stallion spermatozoa (Figure 8A and B).

## Discussion

We monitored the metabolic activity of stallion spermatozoa in the presence of specific metabolites through the label-free fluorescence measurement of NAD(P)H and FAD using flow cytometry. Changes in NAD(P)H and FAD fluorescence in response to different metabolites and combinations were connected with sperm functionality. When stallion spermatozoa were exposed to glucose, NAD(P)H fluorescence increased compared to spermatozoa incubated in the absence of external energy sources. As expected, incubation in the presence of the glucose non-metabolizable analogue 2-deoxiglucose (2DG) did not induce increases in NAD(P)H fluorescence. The increase in NAD(P)H fluorescence in the presence of glucose as the sole energy source is explained through glucose metabolism, in which glucose is broken down to pyruvate, and NAD^+^ is reduced to NADH. In the process, ATP is also produced. Increases in NAD(P)H also occur during the conversion of pyruvate to acetyl-CoA, and subsequently, the TCA cycle produces additional NADH [46]; thus, increases in NAD(P)H fluorescence also reflect lactate and pyruvate metabolism [47]. The spermatozoon is a highly compartmentalized cell, with glycolysis occurring in the cytosol and especially along the flagellum, and the TCA cycle in the mitochondrial matrix [9, 11, 48, 49]. To determine the contribution of other metabolites, stallion spermatozoa were also incubated in the presence of lactate and pyruvate; in the presence of both metabolites, NAD(P)H fluorescence increased. While glucose did not modify FAD fluorescence, lactate and pyruvate induced a significant reduction of FAD fluorescence, showing a global shift of the spermatozoa to a more reduced state. This finding may reflect a preferential mitochondrial sperm metabolism in the presence of lactate and pyruvate; lactate increases the NADH pool after conversion to pyruvate through the action of LDHs [31], and pyruvate is metabolized in the TCA cycle, through the pyruvate dehydrogenase complex (PDC) generating Acetyl CoA and increasing the NADH pool [50]. The decrease in FAD fluorescence can be explained by the reduction of FAD (fluorescent) to FADH₂ (non-fluorescent) at Complex II (SDH) of the ETC. Electrons from FADH₂ are transferred to coenzyme Q, entering the ETC at Complex II—a pathway distinct from NADH, which donates electrons at Complex I. This mechanism supports the observed changes in the ORR, reflecting shifts in mitochondrial redox state.

In our experiments, where spermatozoa were incubated with lactate as the sole exogenous energy source, the synchronous increase in NADH fluorescence and decrease in FAD fluorescence indicate a global shift toward a more reduced redox state [51]. This pattern is consistent with active lactate oxidation, which generates reducing equivalents that can saturate the re-oxidation capacity of the mitochondrial ETC. Such redox behavior highlights the ability of spermatozoa to utilize lactate efficiently, while also suggesting that sustained reliance on this substrate may alter the balance of electron flow and redox homeostasis. This observation was confirmed in experiments designed to study the capacity of stallion spermatozoa to metabolize different substrates, demonstrating a high ability to oxidize lactate but not pyruvate. This indicates that while electron donors are abundant due to active catabolism, the capacity for electron acceptance (re-oxidation by the ETC) may be reaching its limits or is being dynamically regulated in response to the high energy supply. A plausible explanation, in line with previous work from our laboratory, is the operation of an intracellular lactate shuttle in stallion spermatozoa [31]. Since most media and physiological conditions expose spermatozoa to different sources of energy, new experiments were performed. These experiments were conducted using a physiologically balanced salt solution and different sources of energy, as well as various combinations thereof.

Furthermore, functional assays were also performed. While no effects were observed in the percentages of live, motile, and linear motile spermatozoa, sperm velocities, especially the parameter of KE [33], were highly influenced by the energy source or its combinations present in the media. Particularly noteworthy is the effect on KE, which reflects the distance travelled by the spermatozoa per cross of the sperm head along its path, and can be a reflection of both metabolic efficiency and a proxy of hyperactivation [33]. In line with this, mitochondrial functionality estimated through the MAI, was significantly improved in the presence of lactate, and combinations of glucose and lactate and/or pyruvate. Interestingly, both KE and MAI correlated positively with a more reduced redox state as revealed by the NAD(P)H/FAD ratio.

Several other aspects are worth mentioning. The first is that stallion spermatozoa maintain motility and viability in the absence of external energy sources, whenever a balanced salt solution containing Ca^2+^ is provided, confirming previous observations suggesting that spermatozoa dispose of internal sources of energy [52]. Moreover, we previously described the neutral lipid fraction of the stallion spermatozoa; neutral lipids, particularly triacylglycerols and diacylglycerols (DAGs), that may serve predominantly as the source of oxidizable energy [53, 54].

In a separate experiment, we studied the capability of stallion spermatozoa to metabolize glucose, fructose, lactose, pyruvate, lactate and oxoglutarate. We identified glucose, lactate and oxoglutarate as the metabolites preferentially metabolized by stallion spermatozoa, with lactate leading to a more reduced state than glucose. This may represent the action of LDH-B oxidizing lactate to pyruvate, with concomitant production of NADH. This isoenzyme has recently been identified in the stallion spermatozoa in our laboratory, using proteomics and western blotting [31]. The finding of glucose and lactate as the main metabolites oxidized by the stallion spermatozoa is not surprising when the compartmentalization of the stallion spermatozoa is considered [31]. Glucose is preferentially metabolized in the flagella, where the specific sperm isoform of LDH-C, which has high affinity for pyruvate, regenerates NAD^+^ and supports glycolysis. On the contrary, in the mid piece, lactate in the mitochondria may be the preferentially oxidized metabolite; we recently identified the mitochondrial isoform LDH-B; this isoform has a higher affinity for lactate that is converted to pyruvate, producing NADH in the process. The collaboration between glycolysis and oxidative phosphorylation is well known in the stallion spermatozoa [31, 55, 56]. Pyruvate was not preferentially metabolized by the stallion spermatozoa; a similar finding has been recently published in a mouse model [33], and as described in the mouse model, the role of pyruvate may relate to the regulation of the redox environment in the spermatozoa as a mechanism of metabolic regulation. In the stallion spermatozoa, pyruvate contributes to maintaining aerobic glycolysis through the regeneration of NAD^+^, this mechanism is compatible with findings reporting positive effects of pyruvate on stallion spermatozoa [57]. In contrast with mouse spermatozoa, in which lactate is preferentially oxidized, stallion spermatozoa oxidize glucose and lactate; this may relate to the existence of a lactate shuttle in the stallion spermatozoa, but not in the mouse [58, 59]. Indeed, experiments measuring NADH and FAD were performed after 2 h of incubation, whereas the resazurine assay was conducted after 30 min and 1 h. These assays were designed as independent but complementary approaches, each providing meaningful results on their own. While the assays were not intended to be directly combined, future studies integrating these measurements at matched time points would be valuable. In sum, we developed a simple flow cytometry-based redox-metabolic assay; this assay, in combination with the resazurin metabolite oxidation assay, provided interesting outcomes, showing the preferential oxidation of glucose and lactate by the stallion spermatozoa, which reflects the special metabolic compartmentalization and cooperation between glycolysis in the flagella and OXPHOS in the mitochondria. Moreover, this assay may be introduced in the clinical assessment of the spermatozoa, considering that changes in metabolism relate to actual changes in the physiology of spermatozoa [13, 60, 61]. Furthermore, this approach may be especially useful in the design of extenders for sperm conservation and the improvement of media for in vitro fertilization.

## Supplementary Material

Supplementary_Figure_1_ioaf294

Supplementary_Figure_2_ioaf294

Supplementary_Figure_3_ioaf294

Supplementary_materials_ioaf294

## Data Availability

Data are within the paper, raw FCS files are available upon reasonable request to the corresponding author.
