# Supplementary material for: Single-cell metabolic profiling of stallion spermatozoa by flow cytometry using NADH and FAD autofluorescence
Source: Biol Reprod. 2026 Jan 5;114(6):2023–36. doi: 10.1093/biolre/ioaf294 (PMC13273293; doi:10.1093/biolre/ioaf294)
Supplement: Supplementary_Figure_1_ioaf294 [file supplementary_figure_1_ioaf294.docx]

**Supplementary Figure 1. Validation of TMRM staining in stallion spermatozoa.** FCCP served as the negative control, oligomycin as the positive control, and titration with 20–500 nM TMRM defined optimal staining conditions; representative cytometry images illustrate the staining pattern. Statistical significance: *P < 0.05; **P < 0.01; ***P < 0.001; ****P < 0.0001.
