# Supplementary material for: Single-cell metabolic profiling of stallion spermatozoa by flow cytometry using NADH and FAD autofluorescence
Source: Biol Reprod. 2026 Jan 5;114(6):2023–36. doi: 10.1093/biolre/ioaf294 (PMC13273293; doi:10.1093/biolre/ioaf294)
Supplement: Supplementary_Figure_2_ioaf294 [file supplementary_figure_2_ioaf294.docx]

Supplementary Figure 2. Representative cytograms of the label-free flow cytometric determination of NADH and FAD fluorescence. Stallion spermatozoa were processed as described in materials and methods, and the intrinsic fluorescence of NADH and FAD was determined in the presence of different energy sources, including basal (no exogenous energy sources), glucose, lactate, pyruvate, glucose + lactate, and glucose + pyruvate. Cytograms represent concatenated data from 3 stallions, 3 replicates each. High NADH is represented in the lower right quadrant, and high FAD fluorescence is represented in the upper right quadrant.
