# Supplementary material for: Single-cell metabolic profiling of stallion spermatozoa by flow cytometry using NADH and FAD autofluorescence
Source: Biol Reprod. 2026 Jan 5;114(6):2023–36. doi: 10.1093/biolre/ioaf294 (PMC13273293; doi:10.1093/biolre/ioaf294)
Supplement: Supplementary_Figure_3_ioaf294 [file supplementary_figure_3_ioaf294.docx]

Supplementary Figure 3.- Semen samples were incubated in the presence of CCCP (1 μM) and the inhibitor of complex I of the electron transport chain, rotenone (1 μM). As hypothesized, inhibition of the complex I induced a significant increase in NADH fluorescence (Supplementary Figure 3A; *P*=0.0241) while uncoupling the mitochondria caused a substantial reduction in NADH fluorescence (Supplementary Figure 3B; *P*=0.0004). The NADH/FAD and ORR also showed the expected changes with a shift to a more reduced state after inhibition of the electron transport (Supplementary Figure 3D; *P*<0.0001) and to a more oxidized state after mitochondrial uncoupling (Supplementary Figure 3C; *P*<0.0001)
