# Supplementary material for: Single-cell metabolic profiling of stallion spermatozoa by flow cytometry using NADH and FAD autofluorescence
Source: Biol Reprod. 2026 Jan 5;114(6):2023–36. doi: 10.1093/biolre/ioaf294 (PMC13273293; doi:10.1093/biolre/ioaf294)
Supplement: Supplementary_materials_ioaf294 [file supplementary_materials_ioaf294.docx]

**Supplementary Figure 1. Validation of TMRM staining in stallion spermatozoa.** FCCP served as the negative control, oligomycin as the positive control, and titration with 20–500 nM TMRM defined optimal staining conditions; representative cytometry images illustrate the staining pattern. Statistical significance: *P < 0.05; **P < 0.01; ***P < 0.001; ****P < 0.0001.

Supplementary Figure 2. Representative cytograms of the label-free flow cytometric determination of NADH and FAD fluorescence. Stallion spermatozoa were processed as described in materials and methods, and the intrinsic fluorescence of NADH and FAD was determined in the presence of different energy sources, including basal (no exogenous energy sources), glucose, lactate, pyruvate, glucose + lactate, and glucose + pyruvate. Cytograms represent concatenated data from 3 stallions, 3 replicates each. High NADH is represented in the lower right quadrant, and high FAD fluorescence is represented in the upper right quadrant.

Supplementary Figure 3.- Semen samples were incubated in the presence of CCCP (1 μM) and the inhibitor of complex I of the electron transport chain, rotenone (1 μM). As hypothesized, inhibition of the complex I induced a significant increase in NADH fluorescence (Supplementary Figure 3A; *P*=0.0241) while uncoupling the mitochondria caused a substantial reduction in NADH fluorescence (Supplementary Figure 3B; *P*=0.0004). The NADH/FAD and ORR also showed the expected changes with a shift to a more reduced state after inhibition of the electron transport (Supplementary Figure 3D; *P*<0.0001) and to a more oxidized state after mitochondrial uncoupling (Supplementary Figure 3C; *P*<0.0001)
